# Elevation-dependent groundwater control on baseflow in a himalayan catchment: an integrated isotopic–hydrological assessment

**DOI:** 10.1038/s41598-026-49483-2

**Published:** 2026-05-10

**Authors:** Siddharth Arora, Prosenjit Ghosh, Anil V. Kulkarni, Mao-Chang Liang

**Affiliations:** 1 Divecha Centre for Climate Change, Indian Institute of Science, Bengaluru, India; 2 Centre for Earth Sciences, Indian Institute of Science, Bengaluru, India; 3 National Institute of Hydrology, Roorkee, India; 4 Institute of Earth Science, Academia Sinica, Taipei, Taiwan

**Keywords:** Himalayan Catchment, Stable Isotopes, VIC Hydrological Model, Baseflow Contribution, Elevation Dependency, Climate sciences, Environmental sciences, Hydrology, Water resources

## Abstract

Integrating the Variable Infiltration Capacity (VIC) hydrological model with stable isotope tracers, the study establishes a robust method for quantifying baseflow contributions, providing a reliable tool for Integrated Water Resource Management (IWRM) and allowing for cross-validation of simulated groundwater dynamics. Extensive seasonal sampling of river water, springs, and precipitation across elevation gradients was combined with calibrated hydrological simulations. Isotope-based mixing models and VIC outputs show good agreement, revealing a pronounced elevation-dependent increase in baseflow contribution during the dry season. Our study reports that baseflow is responsible for sustaining perennial streamflow,with contribution exceeding 70% at elevations above 1500 m, and up to 97.0 ± 1.5% at higher elevations (> 3000 m) , thus acting as the primary water resource for supporting dry-season water demand. This quantification is critical for ensuring sustainable groundwater resources upstream and managing river water scarcity in downstream regions. The findings in this study directly support India’s Jal Jeevan Mission, Springshed Management, and National Water Policy by providing evidence-based protection for groundwater-fed springs, which are vital to rural drinking water security. This integrated framework allows stakeholders to identify hydrologically sensitive zones and maintain ecological flows amidst changing climate patterns.

**Supplementary Information:**

The online version contains supplementary material available at 10.1038/s41598-026-49483-2.

## Introduction

Baseflow constitutes a critical component of river discharge in Himalayan River systems, particularly during dry seasons when catchment runoff is minimal. The detailed assessment of water resources and components in the hydrological cycle is critical for meeting the water demand and is an important parameter in Integrated Water Resource Management (IWRM). In perennial rivers, baseflow occurs year-round; however, the persistence and stability of subsurface storage becomes especially critical during prolonged dry periods. In glacially and rainfall-fed Himalayan River systems, baseflow sustains streamflow during lean seasons and plays a central role in maintaining water availability for ecosystems, domestic use, and irrigation [] (). Consequently, a robust assessment of baseflow within the hydrological cycle is essential for water-resource sustainability and effective management of available water resources.

One of the major challenges in Himalayan River hydrology is quantifying the contribution of baseflow to total streamflow, as this contribution varies both spatially and seasonally. In Himalayan catchments, river water represents a complex mixture of meltwater, rainfall, and groundwater, each characterised by distinct stable isotope signatures. Stable isotopes of water provide an integrated signal of these hydrological components and enable the separation of individual contributions through conservative isotopic mass-balance approaches^1–3^. During dry periods, groundwater contribution typically dominates streamflow, allowing the identification of groundwater end-member compositions within the river water as admixture.

Recent studies have reported a declining trend in discharge from major Himalayan rivers, attributed to reduced glacial and snowmelt inputs and to excessive groundwater abstraction, leading to a lowering of groundwater tables^4^. These changes have significantly affected dry-season streamflow and pose a serious threat to downstream water security. Increased groundwater withdrawal in mountainous regions exacerbates water scarcity in downstream areas, particularly during the pre-monsoon summer months [36] . Himalayan river systems derive surface water from multiple sources, including direct runoff, snowmelt, glacier melt, and groundwater, with the relative contribution of each varying across space and time. Evaluating the spatial and temporal contribution of groundwater to river discharge is therefore a crucial component of Integrated Water Resources Management (IWRM). Mountain regions are particularly vulnerable to freshwater depletion, largely driven by groundwater loss, which may be further intensified by declining glacial melt and reduced precipitation under changing climatic conditions^5 by end of the century^.

Baseflow represents the groundwater discharge component of streamflow and is a key parameter in the hydrological cycle^6,7^. Accurate quantification of baseflow is essential for water budgeting and resource management, especially in mountainous terrains where baseflow sustains perennial rivers and supports spring-fed water supplies. In this study, baseflow is estimated using the Variable Infiltration Capacity (VIC) hydrological model, in which baseflow drainage is parameterized as outflow from the bottom soil layer following an ARNO-style formulation^8^. The VIC model incorporates both a variable infiltration curve and a baseflow generation curve, with key parameters governing the partitioning of precipitation into infiltration, surface runoff, and baseflow. Since baseflow cannot be directly measured, model-derived estimates require independent validation to ensure scientific robustness.

Stable isotopes of water (δ²H and δ¹⁸O) serve as naturally occurring tracers to quantify the relative contributions of different water sources to streamflow, including baseflow. Reliable isotope-based baseflow estimation necessitates extensive temporal and spatial sampling of stream water, groundwater (springs), and precipitation to capture variability and minimize uncertainty. Isotope-derived baseflow estimates offer an independent and physical approach for validating hydrological model outputs and assessing the reliability of model-based partitioning of streamflow components.

In this study, we integrate hydrological modelling with stable isotope tracer techniques (δ¹⁸O and δ²H) to robustly quantify baseflow contributions to streamflow in a Himalayan River system. This combined approach aims to improve confidence in baseflow estimation and enhance understanding of groundwater – surface water interactions in a data-scarce mountainous region. By coupling isotope mass-balance approaches with process-based hydrological modelling, it is possible to disentangle the relative contributions of groundwater-derived baseflow, direct precipitation runoff, and cryospheric inputs to river discharge across seasonal and spatial scales. Such integrated approaches are invaluable in Himalayan catchments, where complex interactions among monsoonal precipitation, snow and glacier melt, and groundwater storage govern river flow regimes. Previous studies have demonstrated that variations in δ¹⁸O and δ²H in river waters reflect mixing between rainfall, groundwater, and meltwater sources, enabling quantitative separation of streamflow components and improved understanding of watershed hydrodynamics^9–11^. In Himalayan basins, isotopic investigations further reveal that streamflow integrates signals from precipitation, snowmelt, and groundwater recharge, thereby providing an effective framework for assessing seasonal water sources and baseflow dominance in mountainous hydrological systems^12,13^,^38^. Combining isotope tracers with hydrological models therefore offers a powerful method for constraining water residence times, groundwater – surface water interactions, and the resilience of Himalayan river systems to climate variability and hydrological inputs.

**Fig. 1:**
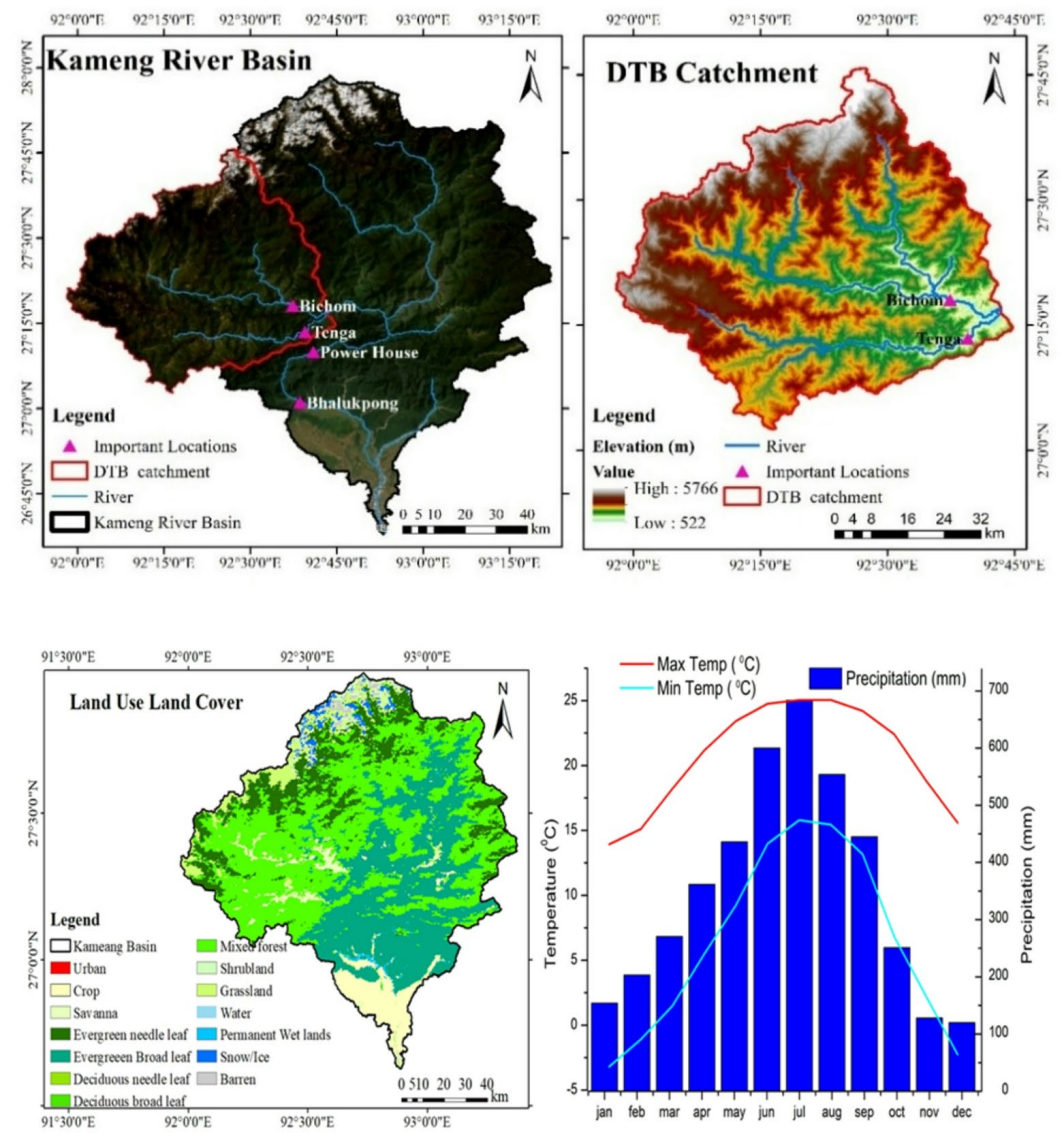
Study area characteristics: location, elevation, LULC and annual precipitation & temperature.

The present study focuses on the Dirang–Tenga–Bichom (DTB) catchment within the Kameng River Basin, located in Arunachal Pradesh, northeastern India. This region has garnered considerable attention owing to the proposed development of multiple small- and medium-scale run-of-the-river hydropower projects. The implementation of these projects necessitates the construction of small reservoirs to regulate short-term fluctuations in streamflow and to stabilise water availability. Located in West Kameng District, Arunachal Pradesh, India. The Kameng river basin lies between 26.5°N and 28°N and 91.75°E and 93.5°E, encompassing an area of 10,798 sq. km. with elevations varying from 53 m at Tezpur to 7,006 m near the Indo-Tibet border, close to the headwaters. The sub-catchments of Dirang, Tenga, and Bichom lie between 27^0^N and 27.9^0^N and 91.9^0^E to 92.75^0^E, encompassing an area of 3463.92 sq. km with elevation varying from 522 m at the confluence of Dirang and Tenga to 5766 m at the highest point within the catchment (Fig. 1). The DTB catchment encompasses an area of 3464 sq.km with elevation ranging from 522 m to a high point of 5766 m. The region receives a high mean annual rainfall of approximately 4,207 mm, with temperatures ranging from a summer maximum of around 25 °C during June and July to a winter minimum of nearly − 3 °C in December and January. Nearly 65% of the annual precipitation occurs over a 150-day period from May to September, coinciding with the Indian Summer Monsoon (ISM). Of the total geographical area (~ 9,058 km²), approximately 83% is covered by dense forest and canopy vegetation (Fig. 1). The catchment is devoid of any glacier and permanent snow-caps. This is a unique setup for experimentation, with minimal input from the cryosphere component other than snowmelt water. Arora et al.^14^,.

The forthcoming construction of hydrological structures such as reservoirs and diversion dams is expected to influence the regional hydrological regime through enhanced evapotranspiration and alterations to the natural river flow patterns^15^. At present, groundwater exploitation in the region accounts for only 0.11% of the total available groundwater resources^43^, indicating that anthropogenic groundwater withdrawal has a negligible impact on regional hydrology during the study period. Furthermore, the evidence of dried-up springs and small channels that once flowed year-round, as observed during the field visit, underscores the need to understand groundwater depletion. Consequently, this study provides the first robust baseline dataset for evaluating future changes in hydrological behaviour associated with infrastructural development and water resource utilisation. In Himalayan and sub-Himalayan landscapes, where a significant proportion of the habitation rely on springs sustained by groundwater, this study presents a robust and transferable framework for quantifying groundwater contributions to streamflow at the catchment scale.

## Methodologies

**Fig. 2:**
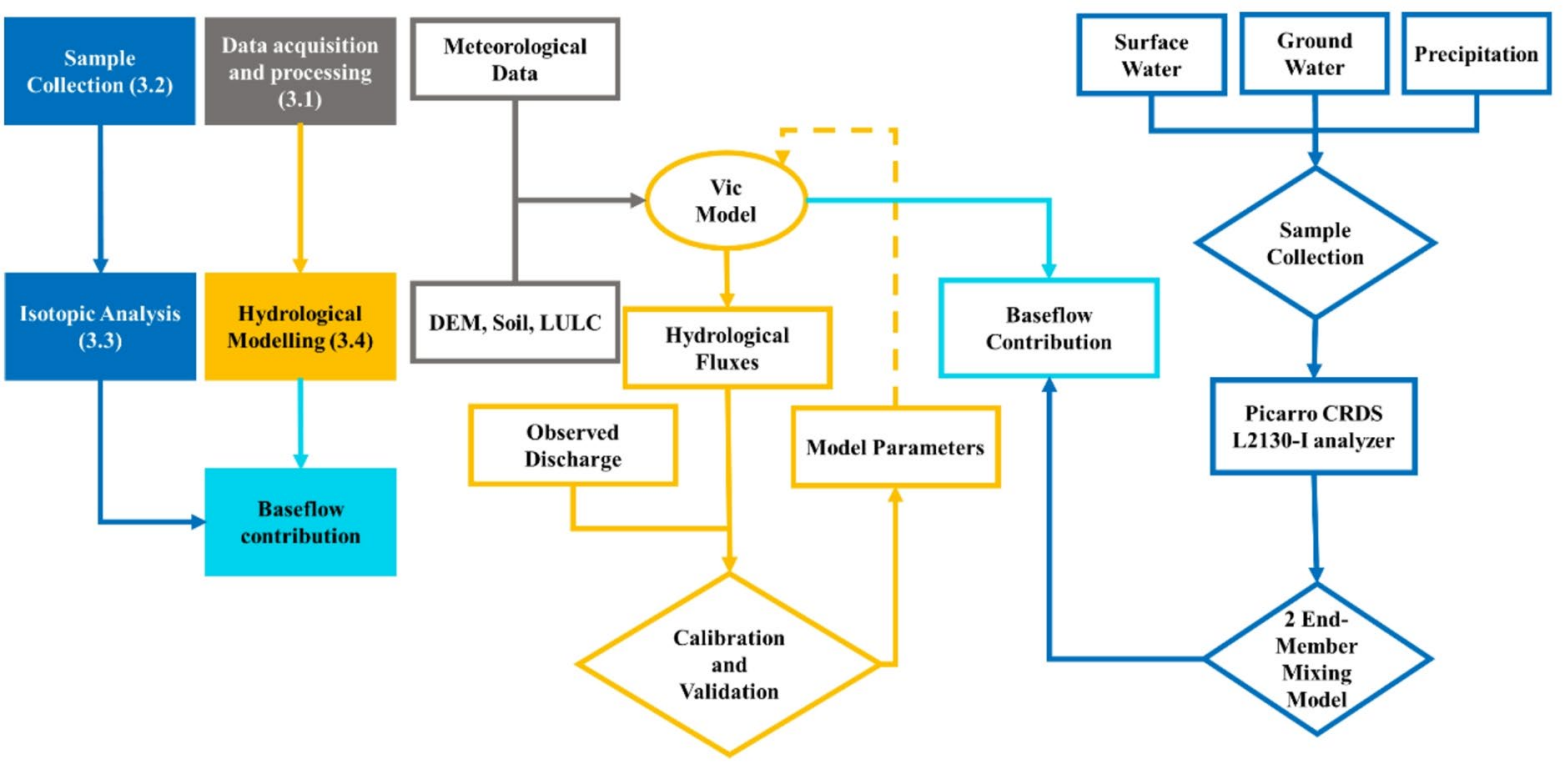
Workflow schematic.

The study is primarily comprises of two components viz. Isotopic analysis and hydrological modelling, which were integrated to develop a framework to estimate baseflow contribution to streamflow in a mountanious catchment (Fig. 2), detailed in the subequent sub-sections.

### Data acquisition and preprocessing

Meteorological forcing for the hydrological model was derived from the ERA5-Land dataset^16^, produced by the European Centre for Medium-Range Weather Forecasts (ECMWF). The ERA5 product provides 3-hourly data at a spatial resolution of 0.1°, including total precipitation, 2-m air temperature, and wind speed, which were aquired for a 45-year period spanning 1979–2024. ECMWF ERA5 datasets have been extensively employed as a surrogate for in-situ meteorological observations in data-sparse mountainous regions such as the Himalayas^17,18^. Owing to the pronounced elevation gradients within the Kameng River catchment, air temperature was downscaled using elevation-difference and lapse-rate-based corrections following Liston and Elder^19^. Precipitation data were spatially re-gridded to a finer resolution of 0.025° to better represent orographic variability across the catchment.

Observed river discharge data at the Bhalukpong gauging station were obtained from the Brahmaputra Basin Organisation (BBO) (erstwhile Brahmaputra & Barak Basin Organization, CWC Shillong)  of the Central Water Commission (CWC), Guwahati, Government of India. The dataset spans the period 2000–2010 and was used for hydrological model calibration and validation, constrained by the limited availability of long-term continuous discharge records in the study basin. Shuttle Radar Topography Mission (SRTM) digital elevation data at 90 m spatial resolution was used to represent topography, delineate the watershed at the specified outlet, and derive terrain attributes including slope, flow direction, and elevation band files required for flow routing and sub-grid scale model execution. Soil information was obtained from the FAO Harmonized World Soil Database (HWSD^20^;) at 1:1,000,000 scale and resampled to the model grid to extract soil texture classes and derive the hydrological and thermal parameters required by the VIC model. Land use and land cover (LULC) data were sourced from the MODIS-based Global Land Cover Climatology dataset^39^ at 0.5 km spatial resolution and re-gridded to the common model resolution. Leaf Area Index (LAI), which controls evapotranspiration and canopy interception processes, was derived from the MODIS LAI product^21^ and similarly reprocessed to match the model grid. All spatial datasets were pre-processed to ensure consistency in spatial resolution, coordinate reference system, and file format, thereby facilitating seamless integration within the VIC modelling framework.

### Sampling of water for isotopic analysis

**Fig. 3:**
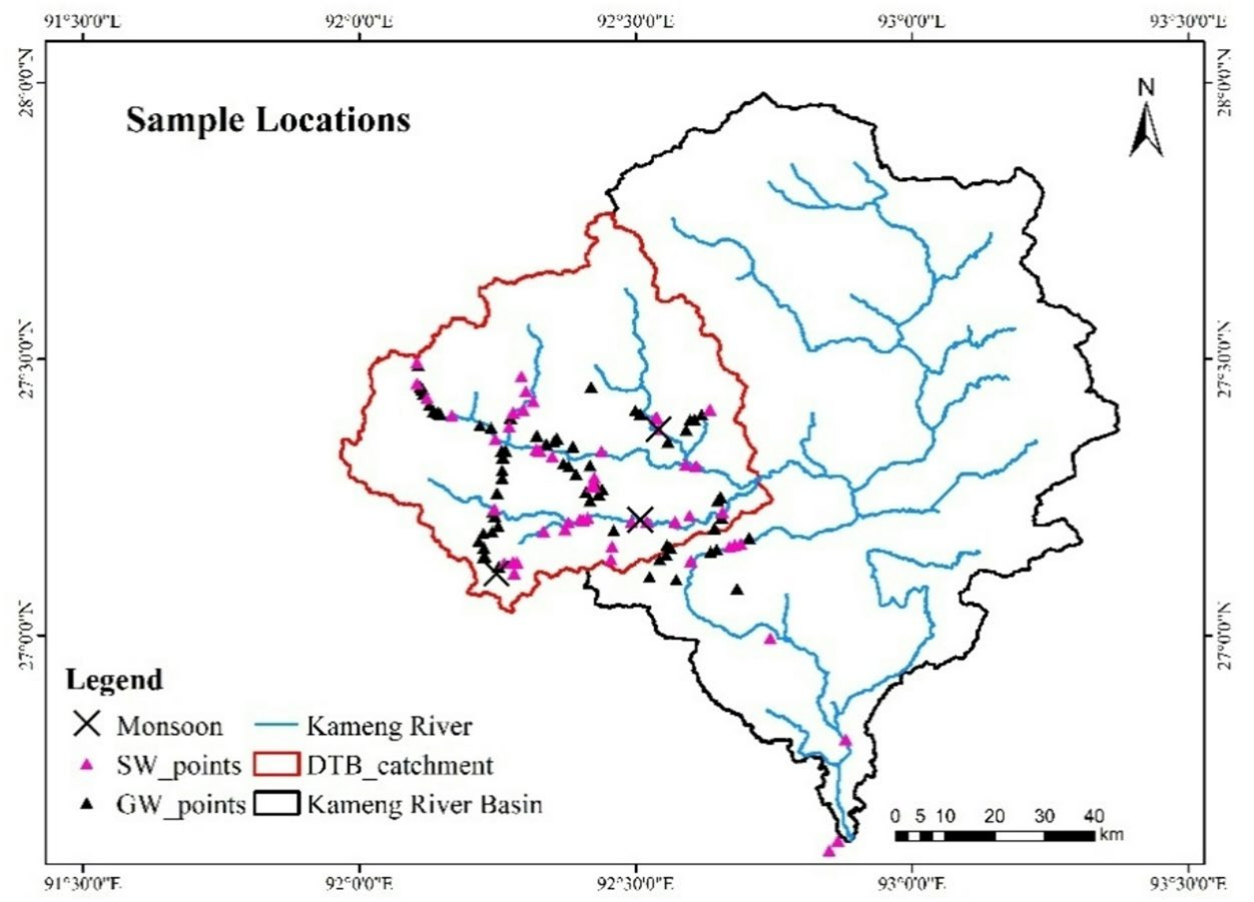
Sample locations.

To investigate the hydrological sources sustaining baseflow, systematic sampling of precipitation, stream water, groundwater, and spring water was conducted along an elevation gradient within the catchment. Groundwater (GW) and surface water (SW) samples were collected from multiple locations across the study area during March 2021, December 2021, and March 2023 (Fig. 3). Sampling campaigns were deliberately scheduled during the pre-monsoon dry season, a period characterized by minimal catchment rainfall and heightened water demand.

The pre-monsoon interval provides a hydrologically stable window for assessing baseflow sources because direct precipitation inputs to the river system are negligible. Under such conditions, sustained stream discharge is predominantly maintained by delayed subsurface contributions, particularly groundwater discharge and spring-fed inputs. Moreover, meltwater contributions from seasonal snowpack are limited during this period in the studied catchment, further enhancing the relative dominance of groundwater-derived flow. Consequently, sampling during the pre-monsoon dry season allows the isotopic and hydrochemical signatures of stream water to more clearly reflect groundwater contributions, thereby providing a robust framework for identifying the primary hydrological sources sustaining baseflow across the elevation gradient. A total of 350 water samples, comprising spring (GW) and river (SW) sources, were collected, providing extensive spatial and temporal coverage. In addition, precipitation samples were collected during the monsoon season at three sites spanning different elevations. Supplementary precipitation samples were also obtained at select locations during field campaigns. All water samples were collected in 60 mL low-density polyethene (LDPE) bottles, ensuring the absence of air bubbles, and were subsequently sealed with paraffin wax and Parafilm to minimise evaporative losses and potential leakage. The geographic coordinates and elevations of each sampling location were recorded using a handheld GPS.

### Isotopic analysis of water samples

The oxygen (¹⁸O) and hydrogen (^2^H) isotopic compositions of the water samples were measured using a Picarro L2130-i cavity ring-down spectroscopy (CRDS) analyser, following the analytical protocol described by Laskar et al.^22^. Isotopic ratios are reported relative to the Vienna Standard Mean Ocean Water (V-SMOW) scale. The standard delta notation of isotopic composition is

1 $$\:\delta\:=\left(\frac{R\mathrm{sample}}{R\mathrm{standard}}-1\right)\mathrm{*}1000{\%}$$

Here, R’s are the ratios of heavy to light isotopes for the standard and sample.

The Global Meteoric Water Line (GMWL) defined by Craig^23^ is given by the Eq.

2 $$\:{\updelta\:}\mathrm{D}=8\mathrm{*}{\updelta\:}\mathrm{1}\mathrm{8}\mathrm{O}+10$$

Commonly, the Local meteoric water line (LMWL) is different from the GWML due to factors like rainfall amount, elevation, evaporation/recycling processes and distance from the moisture source^40^. The deuterium excess defined by Dansgaard^24^ is

3 $$\:\mathrm{d}-\mathrm{e}\mathrm{x}\mathrm{c}\mathrm{e}\mathrm{s}\mathrm{s}={\updelta\:}\mathrm{D}-8\mathrm{*}{\updelta\:}\mathrm{18}\mathrm{O}$$

The d-excess for GMWL is 10‰. A value greater than 10 may indicate recycling of moisture, while a value less than 10‰ indicates local evaporation and influx of moisture.

The calibration process for spectroscopic analysis includes the analysis of six working water standards that were previously calibrated against V-SMOW2 and SLAP2. These six standards have the nominal δ^18^O (δD) values of −1.40 (−10.3) ‰, −3.89 (−28.8) ‰, −6.47 (−48.4) ‰, −10.05 (−66.9) ‰, −12.45 (−88.0) ‰, and − 15.35 (−113.5) ‰. The measurement uncertainties were 0.1‰, 0.5‰, and 0.7‰ for δ^18^O, δD, and d-excess, respectively.

Groundwater contributions to surface water along the elevation gradient of the DTB catchment were quantified using a two-end-member mixing model. The proportional contributions of precipitation (fₚ) and groundwater (fgw) to river water were estimated using isotopic mass balance equations:

4 $$\:\delta\:sw=fp\delta\:p+\:fgw\delta\:gw$$

$$\:fp+fgw=1\:$$.

### Hydrological modelling for baseflow estimation

The VIC hydrological model^25^ was employed to simulate historical hydrological conditions and assess the hydrological fluxes of the Kameng River Basin. VIC is a large-scale, semi-distributed hydrological model widely applied in the Himalayan region for streamflow simulation using both historical meteorological records and future climate projections^14,26,27^. The model can be driven using the outputs from Global Climate Models (GCMs) and operates in either a water balance or an energy balance mode, enabling the estimation of both hydrological and land–atmosphere fluxes. In VIC, the land surface is represented as a grid of uniform cells, each simulated independently. For each cell, the model computes water and energy balance components, with precipitation being the sole atmospheric input for water; lateral flow between cells is not explicitly simulated. This framework allows for detailed cell-level computation while maintaining scalability for large basin studies. To translate simulated runoff into streamflow at specific locations, VIC is coupled with a separate routing model based on the “source-to-sink” concept. This routing scheme uses linearised Saint-Venant equations^28^,^41^ to convolve runoff from individual grid cells and route it to downstream points, producing continuous hydrographs for gauged or project-specific sites. The model was executed at 3 hourly temporal resolution and 0.025^0^ spatial resolution for 1979–2010 with hydrological outputs generated from 2000 onwards to match ground data availability. To stabilise the model and simulate actual soil moisture and snow reservoir in the region before analysing the hydrological outputs, the model was executed for a period of 20-year spin-up from 1979 to 1999, following which analysis was done from 2000 to 2010 at a daily time step. The independent results of simulated grids were processed to depict the status of streamflow and its analysis with observed river discharge data at Bhalukpong, both for the post-calibration and validation periods, respectively. As the present study is designed to estimate the baseflow contribution of streamflow during the lean season, our aim was to accurately simulate and match the low flows during the lean season. For comparison of model output with observed data R^2^, Nash Sutcliffe Efficiency (NSE), refined index of model performance, d_r_^37^ and RMSE-observations standard deviation ratio (RSR) have been used and values of 0.82,0.71,0.78 and 0.54 were obtained respectively at a monthly time step. Based on the model evaluation guidelines as published by Moriasi et al^29^; our model has achieved a “good” performance rating (Figure S2). Due to “classified” nature of data, this publication refrains from publishing the discharge values in the hydrograph. The optimal version of the VIC model framework thus established (calibrated and validated for this specific region) was then used to simulate the hydrological fluxes for the time period coinciding with the sampling time. The calibration process and the parameter values have been provided and included in supplementary Table S3.

As the VIC model estimates hydrological fluxes at grid level, the fluxes were routed at various locations along the elevation to estimate the total flux and flux from surface flow at specified locations. A simple arithmetic operation was used to evaluate the baseflow fluxes and its fraction (F_gw_)

5 $$\:F\:gw=\:\:\frac{Total\:fluxes-Surface\:fluxes}{Total\:Fluxes}$$

## Results

### Effect of hydrological processes on isotopic signatures

The local water lines (LWLs) for surface water (SW) and groundwater (GW) were calculated separately to evaluate the isotopic relationships and infer dominant hydrological processes within the catchment. For surface water, the LWLs derived for March 2021, December 2021, March 2023, and the monsoon period yielded slopes of 7.93 ± 0.3, 8.16 ± 0.2, 8.87 ± 0.1, and 7.85 ± 0.3, with corresponding intercepts of 10.95 ± 2.9, 12.6 ± 2.2, 21.42 ± 1.3, and 7.75 ± 3.0, respectively. For groundwater, the LWLs for March 2021, December 2021, and March 2023 showed slopes of 8.27 ± 0.4, 7.94 ± 0.2, and 8.1 ± 0.1, with intercepts of 12.88 ± 3.9, 9.5 ± 1.8, and 12.26 ± 1.8, respectively. The computed LWLs and the Local Meteoric Water Line (LMWL) are illustrated in Fig. S3, and the corresponding regression parameters are listed in Table S1. The d-excess values further support the isotopic relationships between different water sources. Surface water samples yielded mean d-excess values of 11.5‰ (Mar 2021), 11.3‰ (Dec 2021), 12.8‰ (Mar 2023), and 11.1‰ (monsoon). Groundwater samples exhibited comparable values of 10.6‰, 10.5‰, and 11.5‰ for March 2021, December 2021, and March 2023, respectively. The complete dataset of d-excess values is presented in Table S2 and Fig. S4. Overall, the slopes of the SW and GW LWLs are close to that of the LMWL, and the d-excess values remain consistently similar across precipitation, surface water, and groundwater. This isotopic coherence indicates minimal evaporative modification and suggests that groundwater and surface water largely retain the isotopic signature of monsoon precipitation. These observations imply that monsoon rainfall constitutes the primary recharge source for the regional aquifer system, highlighting the dominant role of seasonal precipitation in sustaining groundwater storage and baseflow in the catchment.

### Baseflow estimation with elevation

Hydrological models simulate the movement and partitioning of water within a catchment by integrating climatic inputs, catchment characteristics, and governing physical processes. As a result, they provide continuous temporal estimates of hydrological fluxes, such as streamflow generation and baseflow contributions, often at daily or sub-daily time steps. However, these estimates are inherently dependent on model structure, parameterization, and calibration procedures, which may introduce uncertainty, particularly in complex mountainous catchments where subsurface flow paths and storage dynamics are poorly constrained. The VIC hydrological model was employed to estimate hydrological fluxes, separately quantifying surface runoff and baseflow contributions along the elevation gradient of the catchment. The fluxes were analysed at a monthly temporal resolution. Model results indicate that baseflow contributions are lowest during the monsoon season, accounting for approximately 20–30% of total streamflow, and increase substantially during the post-monsoon period, reaching a peak contribution of ~ 97% in winter at elevations around 3000 m (Fig. S5). In the absence of permanent snow cover and glaciers within the study region, baseflow plays a critical role in sustaining perennial river discharge, whereas streamflow during the monsoon season is primarily driven by rainfall-induced surface runoff.

**Fig. 4:**
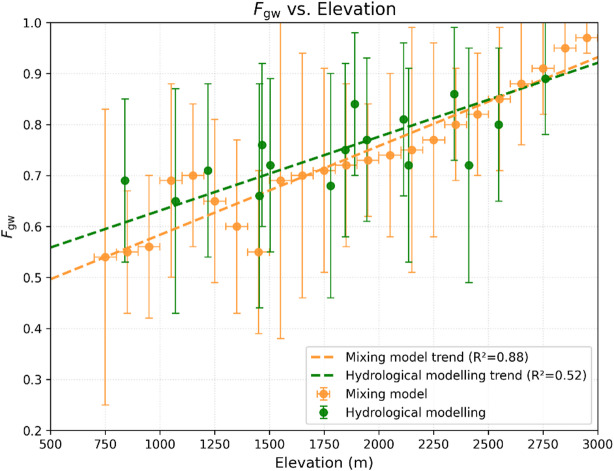
Baseflow contribution along the elevation.

Stable isotopes of water (δ¹⁸O and δ²H) provide an independent natural tracer of hydrological processes and are widely used to identify water sources, mixing relationships, and flow pathways within a catchment. Because isotopic compositions are conserved during water transport (except during phase changes), they retain information about the origin and residence time of water contributing to streamflow. Consequently, isotopic signatures measured in precipitation, groundwater, and surface water can be used to evaluate whether modelled fluxes realistically represent the dominant hydrological sources and pathways. In this context, the isotopic analysis serves as a process-based validation tool for the hydrological model outputs. While the model estimates the magnitude and temporal variability of fluxes such as baseflow or groundwater contributions, isotopic compositions help verify whether these simulated contributions are consistent with the observed mixing patterns between precipitation, groundwater, and stream water. Agreement between model predictions and isotopic evidence strengthens confidence in the simulated hydrological partitioning, whereas discrepancies can reveal limitations in model parameterization or representation of subsurface storage and flow processes. The application of stable isotope signatures, in conjunction with stable isotope-based mixing models, enabled quantification of baseflow contributions, particularly during the dry season. The results indicate a pronounced increase in baseflow contribution with elevation, rising from approximately 50% at lower elevations to nearly 95% at around 3000 m. The elevation-dependent baseflow contributions estimated from the isotopic mixing model and the hydrological model for the sampling period are presented in Fig. 4. The quantification of baseflow contribution was computed at a 100 m elevation interval for isotopic analysis and for a period of 4 years from 2020 to 2024 for the month of March using hydrological modelling. The mean and standard deviation for the datasets as generated were used to plot and evaluate the baseflow contribution.

## Discussions

The role of baseflow as a primary contributor to river discharge in sustaining perennial flow warrants greater attention in future water-resources planning. Baseflow represents a critical component for integrated river basin management and springshed management, particularly in mountainous regions. The present study demonstrates that baseflow is the dominant source sustaining river flow during dry months, whereas its contribution is progressively supplemented and overtaken by rainfall-runoff as the monsoon advances. A reduction in baseflow during critical periods poses a significant threat to streamflow continuity and downstream water availability, potentially leading to water shortages.

This study highlights the effectiveness of combining two independent approaches i.e. computer-based hydrological modelling and field-based isotopic investigations,to quantify elevation-dependent baseflow contributions in data-scarce mountainous catchments. The baseflow fraction estimated from hydrological modelling is independently validated using an isotope-based mass balance approach. The isotopic approach also provides a reliable means to evaluate how climate variability and human activities influence groundwater dynamics in the Himalayan region. By combining isotopic observations with hydrological analysis, it becomes possible to estimate baseflow contributions and identify potential groundwater recharge zones. The approach adopted in this study aligns with the recommendations of the National Institution for Transforming India (NITI) Aayog^30^ and the National Institute of Hydrology^31^, particularly those emphasizing field-based isotope investigations for springshed management.

The results indicate that areas above approximately 2500 m elevation form a critical hydrological zone that functions as a natural reservoir, sustaining lean-season streamflow through spring discharge and groundwater contributions. The framework presented here can be extended to larger spatial domains and longer monitoring periods, and further strengthened by incorporating ensemble-based hydrological modelling. Such an integrated approach would support broader springshed management strategies and contribute to the objectives of the Jal Jeevan Mission.

Isotopic evidence provides valuable insights into the recharge sources of the regional aquifer system. The isotopic composition of groundwater closely resembles that of precipitation, as indicated by the strong agreement between the Local Meteoric Water Line (LMWL) and the Local Water Line (LWL), along with consistent d-excess values. This correspondence highlights the dominant role of monsoon-driven precipitation in groundwater recharge across the region. Consequently, any alteration in monsoon characteristics,such as changes in intensity, spatial distribution, or temporal variabilityis likely to directly influence groundwater recharge and associated baseflow contributions, thereby affecting the seasonal river discharge within the catchment. Multiple studies provide evidence of changing patterns^32–34^ in the North Eastern Region of India which pose a risk to water security in mountainous regions. Such variability can have pronounced local and downstream impacts, particularly on water availability through springs/small streams, ecological flow requirements, and water-dependent economic activities, including irrigation and hydropower generation.

The elevation-dependent variation in baseflow contribution highlights the role of high-elevation aquifers as critical natural reservoirs, which are particularly vulnerable to enhanced drainage and leakage to the river system during dry periods. Macroscale assessments of the Middle Brahmaputra Basin show an annual baseflow contribution of about 40%, with winter contributions rising to 78%^35^. The contribution increases to as much as 97% at higher elevations, particularly during the dry season; however, the VIC model–based estimates show substantially higher uncertainty compared to the isotope-based approach. These results demonstrate the advantage of isotope-based approaches and emphasise the need for isotope monitoring at selected stations to validate VIC-type hydrological model outputs in highly dynamic landscapes such as the Himalayan region. Groundwater replenishment at higher elevations and/or the development of storage reservoirs is critical for sustaining perennial streamflow, particularly in regions lacking permanent snow cover or glaciers. Such streamflow is essential to sustain the ecological, domestic and commercial water use during the dry season. Run-of-the-river projects and small reservoirs, while often considered environmental friendly, can alter natural flow regimes and may alter the groundwater – surface water dynamics. By providing spatially explicit estimates of baseflow contribution along elevation gradients, this approach enables the stake holders to identify hydrologically sensitive zones and to avoid compromising dry season flows and downstream water security. The methodological framework adopted in this study is particularly relevant for regions undergoing rapid hydropower development. The present study used standard hydrologic model evaluation and linked it with the stable isotope tracer-based approach, which provided additional information on water sources, process flux and storage.

## Conclusion and reccomendations

The study provides a clear and consistent evidence that groundwater makes a progressively larger contribution to streamflow within the DTB catchment of the Eastern Himalayas, despite the region receiving high seasonal precipitation. The results indicate that natural recharge alone may not be sufficient to sustain groundwater reserves, particularly at higher elevations where water demand is increasing. Ensuring long-term water security in such mountain systems will therefore require targeted management interventions, including small engineered storage structures, check dams, and the protection and restoration of natural surface water bodies such as lakes and ponds that enhance recharge. By evaluating two widely used baseflow separation approaches, this study demonstrates that hydrological modelling and isotope tracers provide complementary insights into groundwater contributions. However, the VIC model shows increasing uncertainty with elevation, limiting its ability to accurately quantify baseflow in complex Himalayan terrain. In contrast, the isotope-based analysis yields more internally consistent estimates with lower uncertainty, providing stronger evidence for groundwater contributions to streamflow. These findings highlight the value of isotope tracers for constraining hydrological models and improving water balance assessments in mountainous catchments. The outcomes of this work are closely aligned with basin-scale water resource assessment frameworks promoted under national initiatives led by NITI Aayog and the Ministry of Jal Shakti, Govt. of India which emphasize integrated accounting of hydrological components for long-term water security. The results also have direct implications for the Jal Jeevan Mission, where sustainable drinking water supply in remote Himalayan regions depends heavily on groundwater-fed spring systems. Artificial recharge structures should therefore be designed to capture excess monsoon runoff, while water supply infrastructure should be based on lean-season spring discharge to ensure reliability. Furthermore, the study suggests that areas above ~ 2500 m elevation within the DTB catchment likely function as critical recharge zones. Protecting these regions from unregulated land-use change through appropriate policy measures could significantly enhance long-term groundwater sustainability. More broadly, this work presents a transferable framework for quantifying groundwater contributions to streamflow in data-scarce mountain environments. The integration of stable isotope tracers with hydrological modelling provides a robust basis for groundwater-informed river basin management, springshed rejuvenation, and climate-resilient water planning across the Himalayan and sub-Himalayan region.

## Supplementary Information

Below is the link to the electronic supplementary material.

Supplementary Material 1

## Data Availability

The open-source data is available online on respective portals as mentioned in Sect. 2.The discharge data is available with the Central Water Commission on request via a formal channel.The datasets generated during the current study are available from the corresponding author on reasonable request for research purposes only.
