# Supplementary material for: Elevation-dependent groundwater control on baseflow in a himalayan catchment: an integrated isotopic–hydrological assessment
Source: Sci Rep. 2026 May 10;16:21358. doi: 10.1038/s41598-026-49483-2 (PMC13346983; doi:10.1038/s41598-026-49483-2)
Supplement: Supplementary file 1 — Supplementary Material 1 [file 41598_2026_49483_MOESM1_ESM.docx]

**SUPPLEMENTRY**

**Elevation-Dependent Groundwater Control on Baseflow in a Himalayan Catchment: An Integrated Isotopic–Hydrological Assessment**

**Siddharth Arora^1,3 *^, Prosenjit Ghosh^1,2*^, Anil V. Kulkarni^1^, Mao-Chang Liang^4^**

^1^Divecha Centre for Climate Change, Indian Institute of Science, Bengaluru, India (siddhartha1, pghosh, anilkulkarni) @iisc.ac.in; [siddarora.civil@gmail.com](mailto:siddarora.civil@gmail.com);)

**^2^** Centre for Earth Sciences, Indian Institute of Science, Bengaluru, India

^3^National Institute of Hydrology, Roorkee, India ([siddarora.civil@gmail.com](mailto:siddarora.civil@gmail.com));

^4^Institute of Earth Science, Academia Sinica, Taipei, Taiwan (mcl@gate.sinica.edu.tw)

Corresponding author: Siddharth Arora (siddhartha1@iisc.ac.in; siddarora.civil@gmail.com)

Prosenjit Ghosh (pghosh@iisc.ac.in)

| 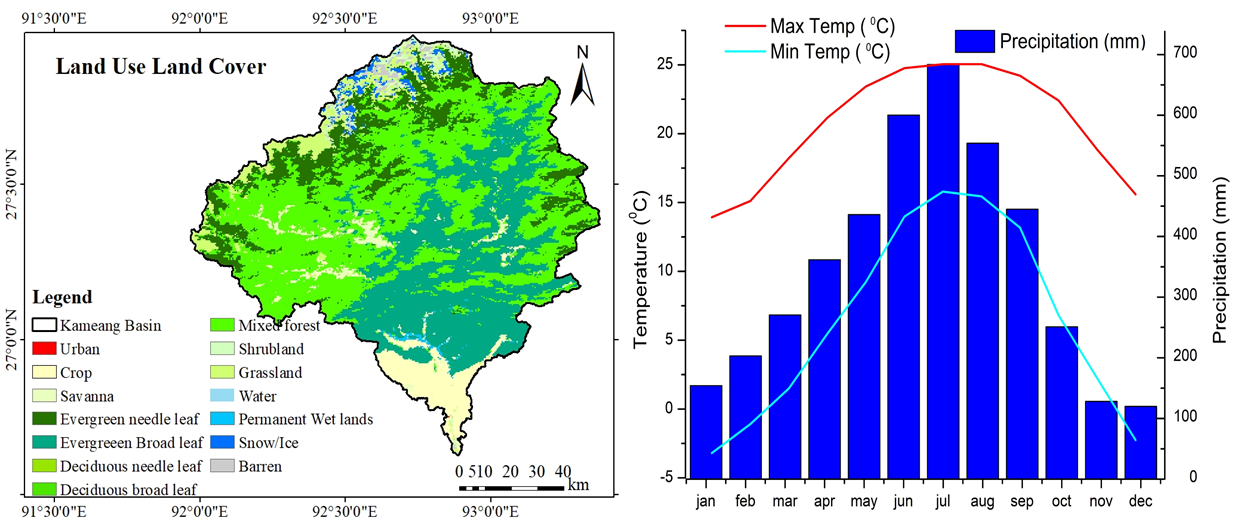 |
| --- |

Figure S1: Land Use Land Cover and Meteorological profile of the study region


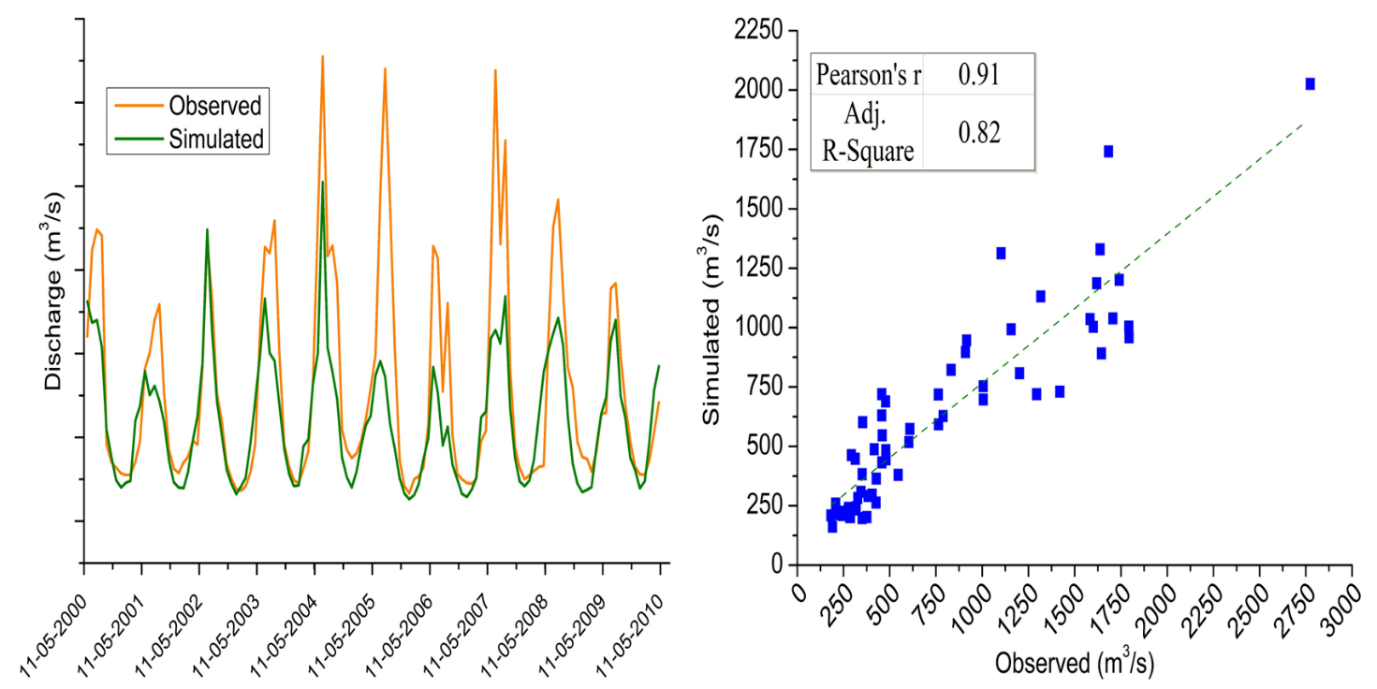


Figure S2: Performance of Model


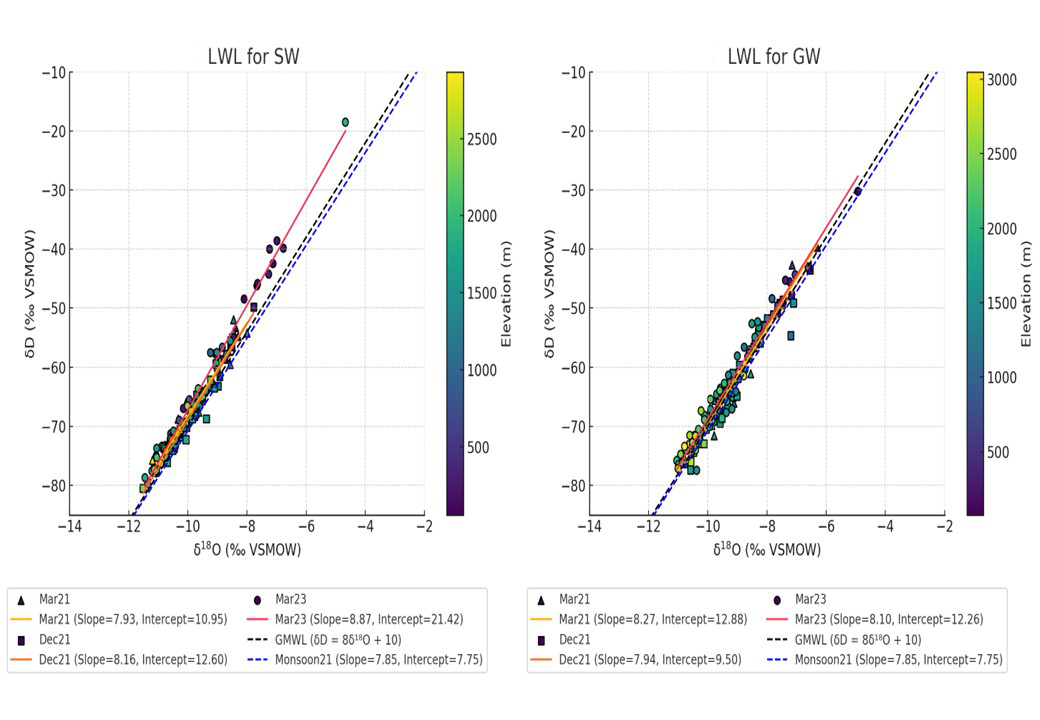


Figure S3: Local Water Lines for SW & GW

Table S1: Values for slope and intercept of LMWL

| **Season** | **Source** | **Slope** | **Intercept** |
| --- | --- | --- | --- |
| Mar21 | SW | 7.93±0.3 | 10.95±2.9 |
| Mar21 | GW | 8.27±0.4 | 12.88±3.9 |
| Monsoon | Rainfall | 7.85±0.3 | 7.75±3.0 |
| Dec21 | SW | 8.16±0.2 | 12.6±2.2 |
| Dec21 | GW | 7.94±0.2 | 9.5±1.8 |
| Mar23 | SW | 8.87±0.1 | 21.42±1.3 |
| Mar23 | GW | 8.1±0.1 | 12.26±1.8 |

| 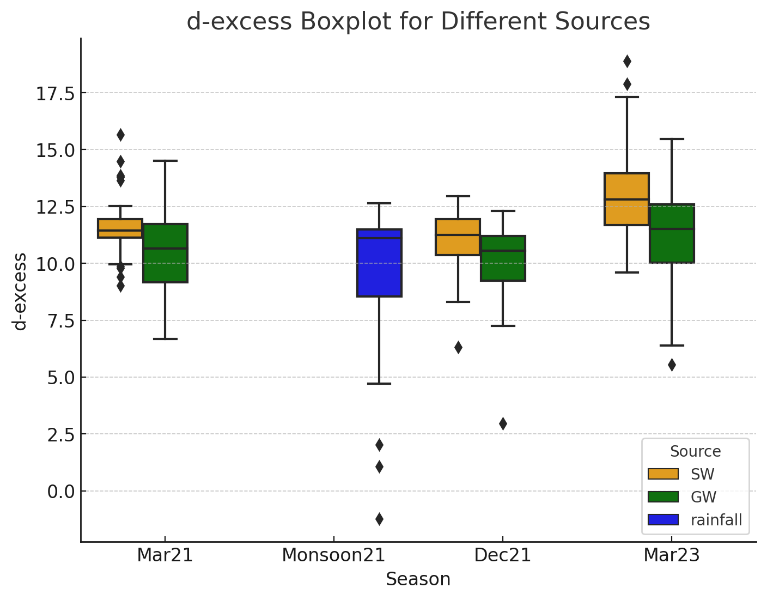 |
| --- |

Figure S4: d-excess for different time intervals for Surface, Ground Water and monsoon

Table S2: Values for d-excess for various sources.

| **Season** | **Source** | **Median** | **Std Dev** |
| --- | --- | --- | --- |
| Mar-21 | SW | 11.5 | 1.4 |
| Mar-21 | GW | 10.6 | 2.1 |
| Monsoon21 | rainfall | 11.1 | 4.0 |
| Dec-21 | SW | 11.3 | 1.3 |
| Dec-21 | GW | 10.5 | 1.7 |
| Mar-23 | SW | 12.8 | 1.9 |
| Mar-23 | GW | 11.5 | 1.9 |

**
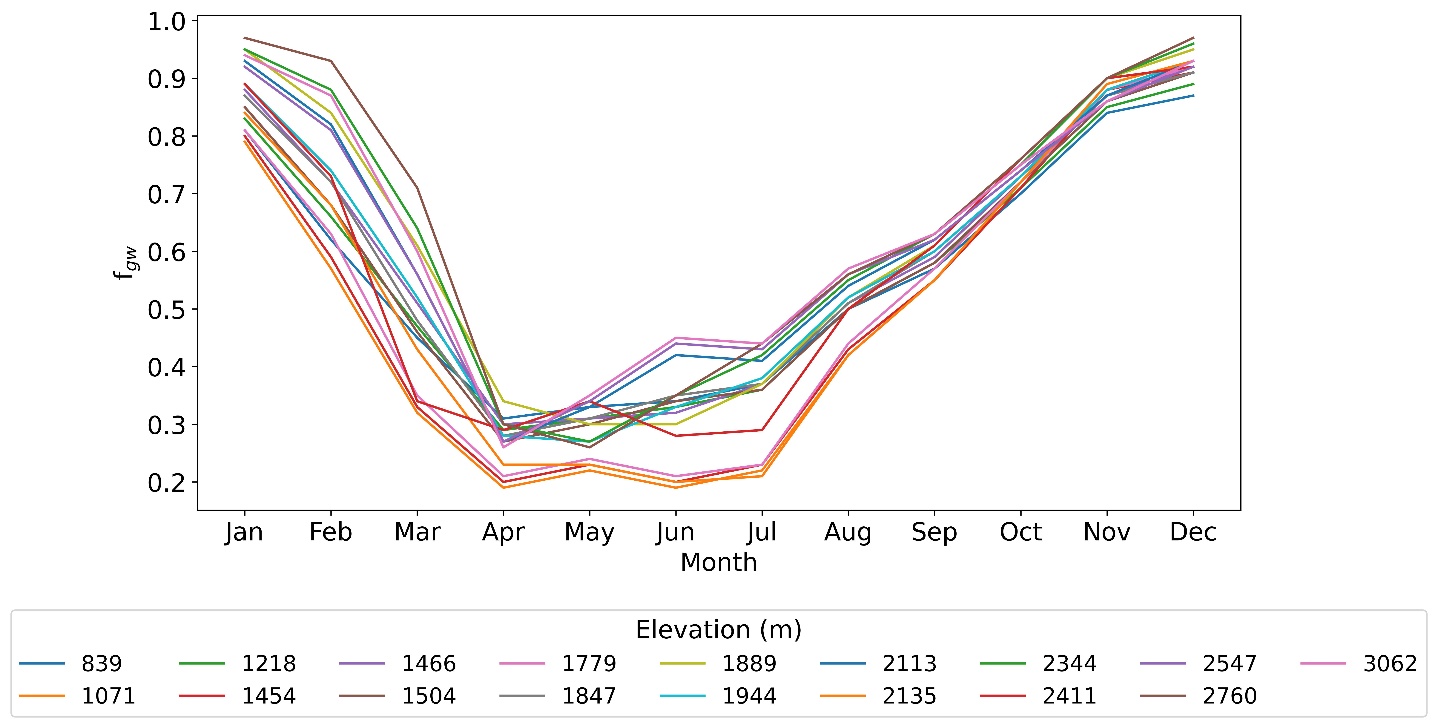
**

Figure S5: Baseflow contribution on a monthly scale at various elevations

Table S3: Calibration Values for Vic Model

Calibration of the VIC hydrological model is the process of adjusting the model parameters such that simulated hydrological behavior agrees with observed streamflow. The calibration process undertakes variation of a set of soil parameters, most commonly the variable infiltration parameter (b_infilt), the baseflow parameters Ds, Dsmax, and Ws, and, soil layer depth. These parameters strongly control how rainfall is partitioned into various hydrological fluxes, viz. infiltration, surface runoff, soil moisture storage, and baseflow generation. The calibration process is carried out with parameters adjusted within physically realistic ranges. The calibration and validation periods are divided along the available ground record (discharge data), so that model performance is tested independently rather than over the full dataset. In practice, calibration is performed iteratively by running the model, comparing simulated and observed hydrographs using performance measures such as NSE, RMSE, or any other suitable performance metric. The parameter values are refined until acceptable agreement is achieved. Overall, VIC calibration aims to obtain a parameter set that is both hydrologically realistic and capable of reproducing observed hydrological fluxes under varying hydroclimatic conditions. The parameters varied in this study are listed below:

| **S.No** | **Variable** | **Range of Value** | **Calibrated Value** |
| --- | --- | --- | --- |
| 1. | Infilt | 0.001–0.4 | 0.25 |
| 2. | Ds | 0.001–1 | 0.5 |
| 3. | Ws | 0.5–0.9 | 0.5 |
| 4. | Depth of soil layer 1 | 01-0.4 | 0.2 |
| 5. | Depth of soil layer 2 | 0.1-1.5 | 1.5 |
| 6. | Depth of soil layer 3 | 0.1-1.5 | 1.5 |
| 7. | Resid_moist | 0.001 To [1- (bulk density/soil density)] | 0.1 |

For further information, the readers may see <https://vic.readthedocs.io/en/master/Documentation/Drivers/Classic/SoilParam/>
